# Hepatoprotective Effect of Aqueous Extract from the Seeds of *Orychophragmus violaceus* against Liver Injury in Mice and HepG2 Cells

**DOI:** 10.3390/ijms18061197

**Published:** 2017-06-15

**Authors:** Xiaowei Huo, Chenqi Liu, Li Gao, Xudong Xu, Nailiang Zhu, Li Cao

**Affiliations:** 1 Institute of Medicinal Plant Development, Chinese Academy of Medical Sciences and Peking Union Medical College, Beijing 100193, China; huoxiaoweiforever@163.com (X.H.); chenqiliu1@163.com (C.L.); gli1986@163.com (L.G.); xdxu@implad.ac.cn (X.X.); 2 Research Center on Life Sciences and Environmental Sciences, Harbin University of Commerce, Harbin 150076, China

**Keywords:** *Orychophragmus violaceus*, epigoitrin, hepatoprotective effect, anti-inflammation, antioxidant

## Abstract

*Orychophragmus violaceus* (*O. violaceus*) is a kind of edible wild herb in north China and its seeds have medical potential, however, the effect of *O. violaceus* seeds on liver injury and the mechanism of action remains poorly understood. Thus, the purpose of the present study is to investigate the effect of *O. violaceus* seeds on liver injury and further explore the molecular mechanism of the beneficial effects using aqueous extract from the seeds of *O. violaceus* (AEOV). Mice were orally administrated with saline, AEOV, and biphenyldicarboxylate for 4 days, and were then injected subcutaneously with 0.1% carbon tetrachloride (CCl_4_) dissolved in corn oil. Sixteen hours later, mice were sacrificed and blood samples were collected. Then, the serum was separated and used for biochemical assay. Livers were excised and were routinely processed for histological examinations. Enzyme activities and protein levels in liver homogenates were detected using commercial kits or by western blot analysis. Additionally, the hepatoprotective effect of AEOV in vitro was evaluated using epigoitrin, the major alkaloid compound isolated from AEOV. We found that AEOV attenuated liver injury induced by CCl_4_ as evidenced by decreased levels of alanine aminotransferase (ALT) and aminotransferase (AST) in serum, improvement of liver histopathological changes, and substantial attenuation of oxidative stress and inflammation via regulation of nuclear factor-erythroid 2-related factor-2 (Nrf2) and nuclear factor κB (NFκB) pathways. These effects of AEOV were comparable to that of biphenyldicarboxylate which was commonly used as a hepatoprotective reference. Moreover, pretreatment of HepG2 cells with epigoitrin improved cell viability, decreased lactate dehydrogenase (LDH) and malondialdehyde (MDA) levels, increased superoxide dismutase (SOD) and glutathione peroxidase (GSH-Px) activity, attenuated the NFκB pathway, and elevated the Nrf2 pathway after exposure to H_2_O_2_. These results suggest that AEOV could effectively prevent CCl_4_-induced liver injury in mice via regulating the Nrf2 and NFκB pathways, and reveal the cytoprotective effects of epigoitrin against H_2_O_2_-induced oxidative stress in HepG2 cells.

## 1. Introduction

Liver injury, generally caused by viral hepatitis, non-alcoholic steatohepatitis, alcohol abuse, and drug intoxication [1,2], has severe consequences for metabolism, detoxification, immune response, and antimicrobial defense because the liver is involved in almost all vital biological processes. The mechanism of liver injury is not well established, however, emerging evidence suggests that multiple mechanisms are postulated to be involved in the development of liver injury such as oxidative stress, dysfunction of intracellular targets, and inflammation, as well as complex interactions between alcohol metabolism, multiple cytokines, and the innate immune system [3,4,5]. Oxidative stress and inflammatory action, based on the involvement of the transcription factor nuclear factor-erythroid 2-related factor-2 (Nrf2) and transcription factor nuclear factor κ B (NFκB), have gained increasing attention largely due to their critical roles in the initiation and progression of liver injury [6,7,8]. Recent studies have suggested that the activation of nuclear factor-erythroid 2-related factor-2–antioxidant response element (Nrf2–ARE) signaling can confer protection to normal cells or tissues by preventing free radical stress [9,10,11]. Additionally, suppression of the NFκB pathway has been found to be essential for the controlling of the expression of pro-inflammatory cytokines which, otherwise, may contribute to tissue damage [12,13]. In this regard, we hypothesize that approaches and drugs successfully used in controlling oxidative stress and inflammation may help protect against liver injury.

In recent years, a number of drugs with anti-oxidative and anti-inflammatory properties have been evaluated as hepatoprotective agents, however, they generally have been proven to be nonspecific and exhibited limited efficacy in the therapy of liver injury [14,15]. Herein, it is necessary to explore more potential agents for the treatment of liver injury, and natural products derived from traditional Chinese herbal medicines may provide alternative treatment options for liver injury. Actually, numerous studies have emphasized the use of natural products for the prevention and therapy of liver injury because of their safety and efficacy as an alternative remedy compared with chemically synthesized drugs [16]. *Orychophragmus violaceus*, also called Eryuelan in China, is a kind of edible wild herb in north China which is rich in linoleic acid favorable for the body. This species has been reported to be valuable due to its high seed yield potential and good quality of seed oil [17]. The seeds of *O. violaceus* also have potential for the treatment of liver disease [18], however, the effect of *O. violaceus* seeds for liver injury induced by CCl_4_ is not clear, and the underlying mechanism of the potential effect remains to be elucidated. 

The current study was, therefore, designed to evaluate the hepatoprotective effect of aqueous extract from the seeds of *O. violaceus* (AEOV) both in vivo and in vitro. In this study, the mouse model of CCl_4_-induced hepatic injury was established to investigate the hepatoprotective effect of AEOV in vivo and elucidate the underlying mechanisms. Furthermore, the hepatoprotective effect of epigoitrin in vitro was evaluated using epigoitrin in H_2_O_2_-induced HepG2 cells. The results showed that the acute liver injury induced by CCl_4_ treatment was significantly attenuated by AEOV treatment compared with untreated mice as determined by the reduced serum levels of aspartate aminotransferase (AST) and alanine aminotransferase (ALT), and less severe liver injury. Epigoitrin, the major alkaloid compound isolated from AEOV, exhibited a strong cytoprotective effect against H_2_O_2_-induced oxidative damage in HepG2 cells. With regard to the possible mechanism, our data revealed that AEOV ameliorated liver injury by decreasing oxidative damage and inhibiting the production of pro-inflammatory cytokine tumor necrosis factor (TNF)-α via regulation of Nrf2 and NFκB signaling pathways. The cytoprotective effect of epigoitrin was also associated with Nrf2 and NFκB pathways. These results highlight the potential of AEOV in the treatment of liver injury.

## 2. Results

### 2.1. High Performance Liquid Chromatography (HPLC) Analysis and Composition of AEOV

**Figure 1:**
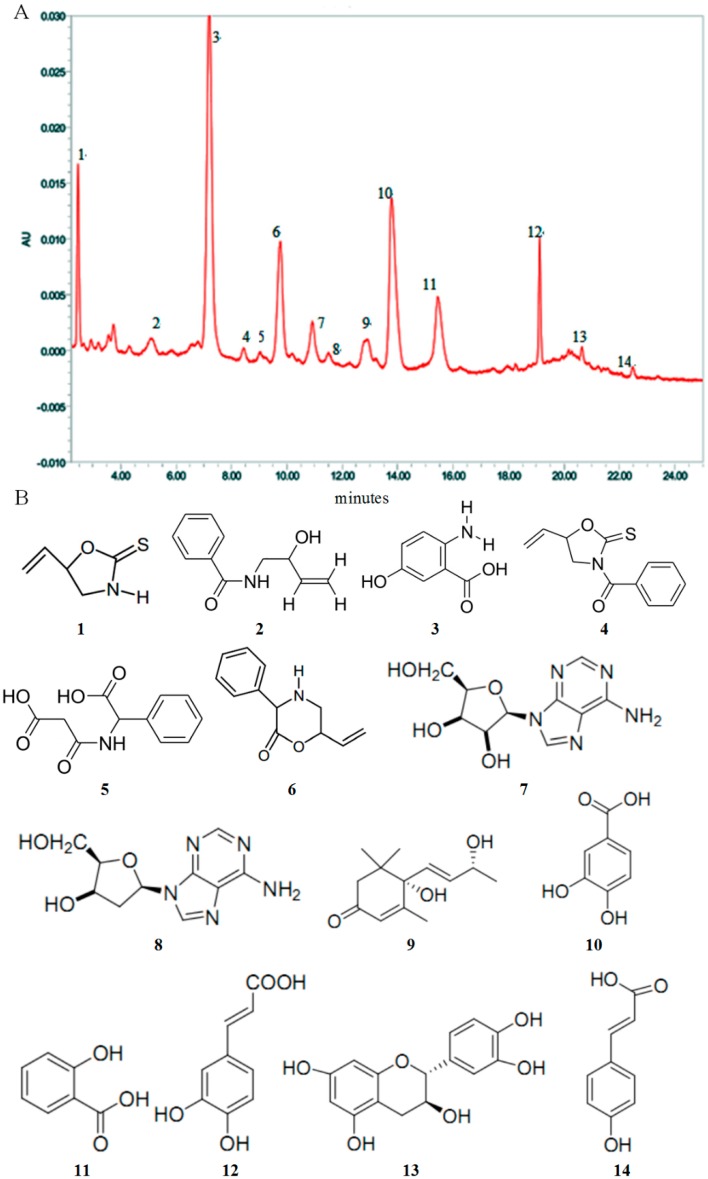
(**A**) The chromatographic profile of aqueous extract from *Orychophragmus violaceus* (AEOV) analyzed by HPLC; (**B**) Chemical structure of the main constituents of AEOV.

The chromatographic profiles of the components of the AEOV were analyzed using high performance liquid chromatography (HPLC) as shown in Figure 1A. Data in Figure 1B show the chemical structure of some exact composition of the mixture. Compounds 1–14 were identified to be epigoitrin (1); *N*-2-hydroxy-3-butenyl-benzamide (2); 2-Amino-5-hydroxybenzoic acid (3); *N*-benzoyl-epigoitrin (4); α-[(2-carboxyacetyl) amino]-benzeneacetic acid (5); 3-phenyl-6-vinylmorpholin-2-one (6); adenosine (7); 2′-deoxy adenosine (8); blumenol A (9); protocatechuic acid (10); salicylic acid (11); caffeic acid (12); (+)–catechin (13); and *p*-coumaric acid (14).

### 2.2. Hepatoprotective Effect of AEOV against CCl_4_-Induced Liver Injury

**Figure 2:**
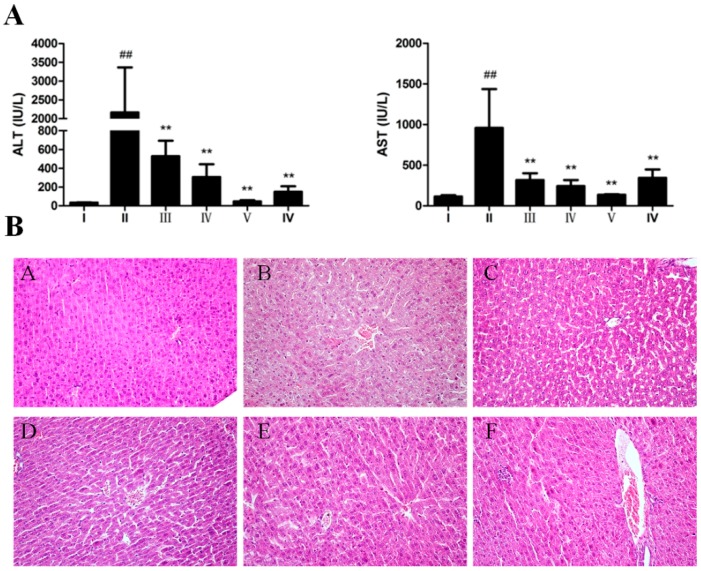
The effect of AEOV on CCl_4_-induced liver injury. (**A**) Serum levels of alanine aminotransferase (ALT) and aminotransferase (AST). Groups are as follows. І: saline; ІІ: saline + CCl_4_; ІІІ: AEOV 125 mg/kg + CCl_4_; ІV: AEOV 250 mg/kg + CCl_4_; V: AEOV 500 mg/kg + CCl_4_; VІ: biphenyldicarboxylate 150 mg/kg + CCl_4_. Values are the means ± standard error of the mean (SEM) of three independent experiments. ## *p* < 0.01 versus normal control group, ** *p* < 0.01 versus CCl_4_-treated group; (**B**) Hematoxylin and eosin (H&E) staining of livers. Mice were treated with saline, AEOV (125, 250, and 500 mg/kg), and biphenyldicarboxylate 150 mg/kg for 4 days, and were then injected subcutaneously with 0.1% CCl_4_ dissolved in corn oil. Sixteen hours later, mice were sacrificed and livers were excised and stained with hematoxylin and eosin (400×). Groups are as follows. A: saline; B: saline + CCl_4_; C: AEOV 125 mg/kg + CCl_4_; D: AEOV 250 mg/kg + CCl_4_; E: AEOV 500 mg/kg + CCl_4_; F: biphenyldicarboxylate 150 mg/kg + CCl_4_.

We established animal model of hepatic injury by intraperitoneal injection of CCl_4_ in Balb/c mice. As shown in Table 1, CCl_4_ injection and AEOV administration had no significant effect on body weight and liver index. Biochemical indicators including AST and ALT were detected in all treatment groups to evaluate the hepatoprotective effect of AEOV. As shown in Figure 2A, serum levels of AST and ALT, which were commonly used as biomarkers for liver injury, were significantly elevated in mice injected with CCl_4_ compared to the control group. Fortunately, AEOV treatment dramatically reduced both of these two indicators. The hepatoprotective effect of AEOV was also assessed by morphological observation of hematoxylin and eosin (H&E)-stained liver tissues. As shown in Figure 2B, histological examination results were well consistent with that of the biochemical parameters. Multifocal hepatic parenchymal necrosis with inflammatory cell infiltration was observed in CCl_4_-induced mice. AEOV and biphenyldicarboxylate treatment significantly ameliorated the degree of hepatic parenchymal necrosis and inflammatory cell infiltration was also attenuated. 

### 2.3. Anti-Oxidative Effect of AEOV on Liver Tissues 

**Figure 3:**
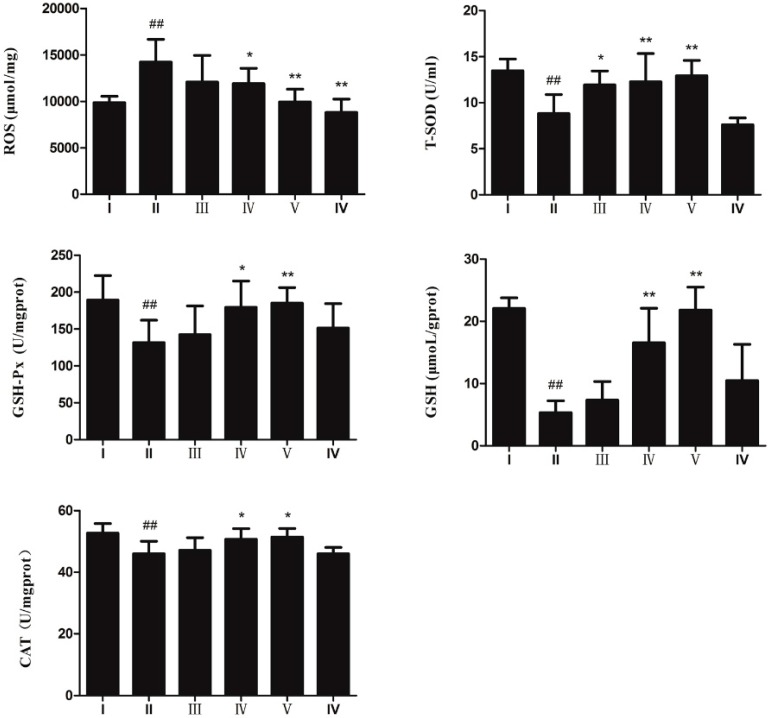
Levels of reactive oxygen species (ROS), superoxide dismutase (T-SOD), glutathione (GSH), glutathione peroxidase (GSH-Px), and catalase (CAT) in liver tissues. Groups are as follows. І: saline; ІІ: saline + CCl_4_; ІІІ: AEOV 125 mg/kg + CCl_4_; ІV: AEOV 250 mg/kg + CCl_4_; V: AEOV 500 mg/kg + CCl_4_; VІ: biphenyldicarboxylate 150 mg/kg + CCl_4_. Values are the means ± SEM of three independent experiments. ## *p* < 0.01 versus normal control group, ** *p* < 0.01, * *p* < 0.05 versus CCl_4_-treated group.

Figure 3 shows that CCl_4_ administration markedly increased the hepatic reactive oxygen species (ROS) level. The AEOV pre-treatment group significantly ameliorated the production of ROS in liver tissues. Anti-oxidative enzyme activity was also tested to evaluate the anti-oxidative effect of AEOV. Superoxide dismutase (T-SOD), catalase (CAT), glutathione peroxidase (GSH-Px), and glutathione (GSH) levels in liver homogenates were significantly decreased in the CCl_4_-treated mice (group ІІ) relative to that of the control group (group І). However, the administration of AEOV significantly reversed CCl_4_-induced decrease of T-SOD, CAT, GSH-Px, and GSH, thus partially suggesting its anti-oxidative effect on the CCl_4_-induced model.

**Figure 4:**
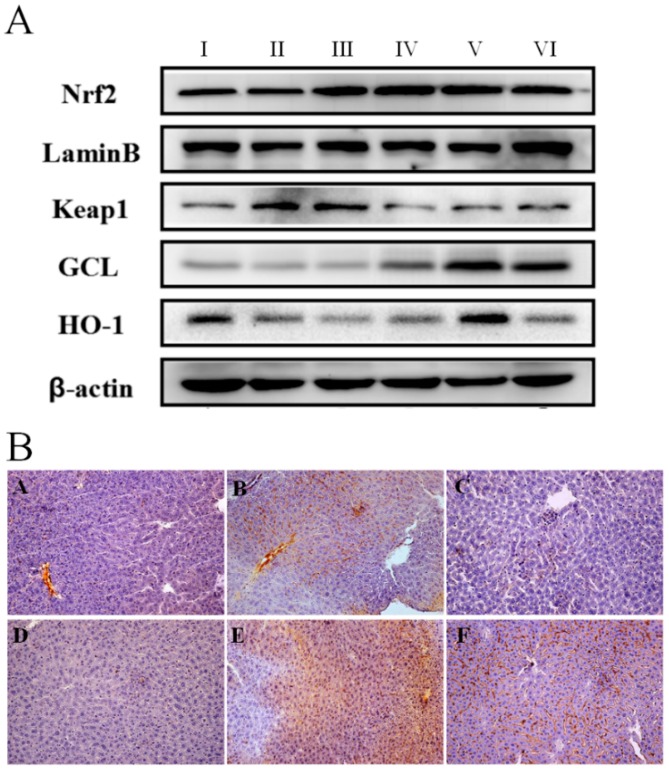
Anti-oxidative effect of AEOV on liver tissues. (**A**) Liver tissues were collected and used to analyze the expression of nuclear factor-erythroid 2-related factor-2 (Nrf2), Kelch-like ECH-associated protein 1 (Keap1), heme oxygenase (HO), and glutamate cysteine ligase (GCL) by western blot analysis. Groups are as follows. І: Saline; ІІ: saline + CCl_4_; ІІІ: AEOV 125 mg/kg + CCl_4_; ІV: AEOV 250 mg/kg + CCl_4_; V: AEOV 500 mg/kg + CCl_4_; VІ: biphenyldicarboxylate 150 mg/kg + CCl_4_; (**B**) Immunohistochemical staining of Nrf2 in liver tissues (400×). Groups are as follows. A: saline; B: saline + CCl_4_; C: AEOV 125 mg/kg + CCl_4_; D: AEOV 250 mg/kg + CCl_4_; E: AEOV 500 mg/kg + CCl_4_; F: biphenyldicarboxylate 150 mg/kg + CCl_4_.

We next sought to determine possible mechanisms for the onset of antioxidant by examining the effect of AEOV on Nrf2 signaling. Results presented in Figure 4A show that AEOV administration significantly down-regulated expression of Kelch-like ECH-associated protein 1 (Keap1), an inhibitor of Nrf2, substantially enhanced accumulation of Nrf2 in nuclei, and markedly increased expression of glutamate cysteine ligase (GCL) and heme oxygenase (HO)-1. Moreover, immunohistochemical examination confirmed the increased expression of Nrf2 in liver tissues after AEOV administration (Figure 4B), which was consistent with the results of western blot. These data strongly demonstrated the anti-oxidative property of AEOV in vivo via the activation of Nrf2 pathway by activating Nrf2/inactivating Keap1 accompanied with the elevation of anti-oxidative enzyme activity.

### 2.4. Anti-Inflammatory Effect of AEOV on Liver Tissues 

**Figure 5:**
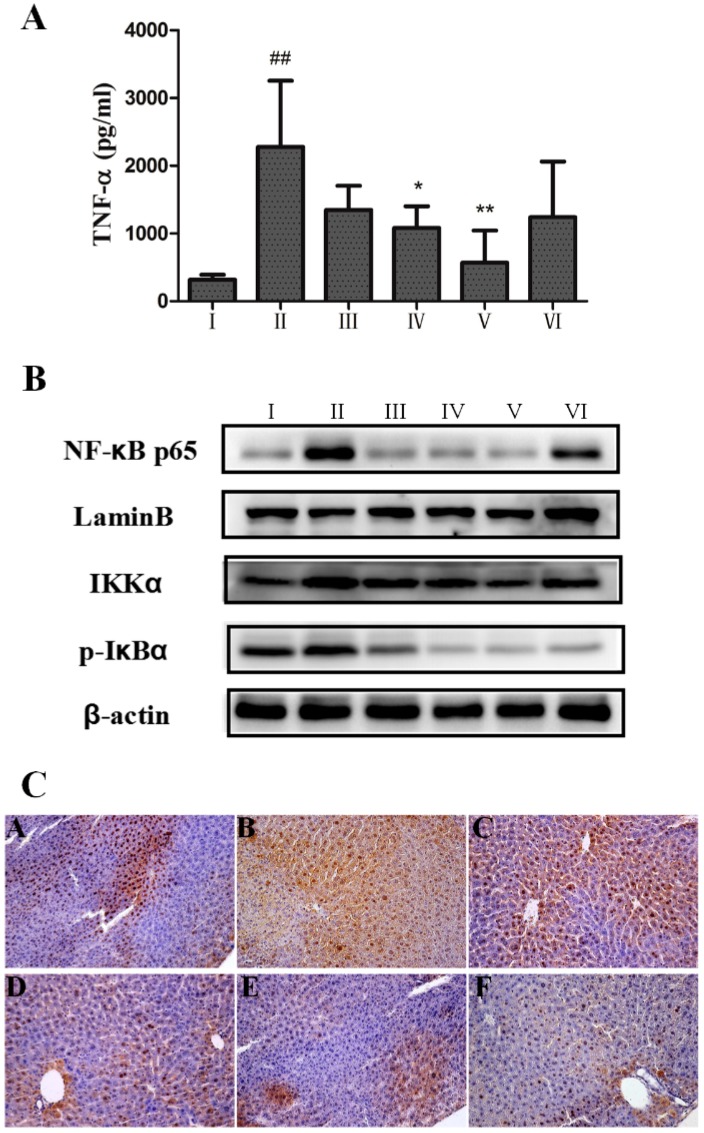
Anti-inflammatory effect of AEOV on liver tissues. (**A**) Serum tumor necrosis factor (TNF)α level. Groups are as follows. І: saline; ІІ: saline + CCl_4_; ІІІ: AEOV 125 mg/kg + CCl_4_; ІV: AEOV 250 mg/kg + CCl_4_; V: AEOV 500 mg/kg + CCl_4_; VІ: biphenyldicarboxylate 150 mg/kg + CCl_4_. Values are the means ± SEM of three independent experiments. ## *p* < 0.01 versus normal control group, ** *p* < 0.01, * *p* < 0.05 versus CCl_4_-treated group; (**B**) Western blot analysis of nuclear factor κ B (NFκB), IκB kinase α (IKKα), and phosphorylated inhibitory factor kappaB-alpha (p-IκBα) in liver tissues. Nuclear proteins were used to test NFκB levels. Groups are as follows. І: saline; ІІ: saline + CCl_4_; ІІІ: AEOV 125mg/kg + CCl_4_; ІV: AEOV 250 mg/kg + CCl_4_; V: AEOV 500 mg/kg + CCl_4_; VІ: biphenyldicarboxylate 150 mg/kg + CCl_4_. (**C**) Immunohistochemical staining of NFκB in liver tissues. Groups are as follows. A: saline; B: saline + CCl_4_; C: AEOV 125 mg/kg + CCl_4_; D: AEOV 250 mg/kg + CCl_4_; E: AEOV 500 mg/kg + CCl_4_; F: biphenyldicarboxylate 150 mg/kg + CCl_4_.

It is well known that modulation of the pro-inflammatory response plays a critical role in liver injury. Consistently, our results in Figure 5A showed that the level of pro-inflammatory cytokine TNF-α was significantly increased after CCl_4_ injection. AEOV significantly attenuated CCl_4_-induced increase of TNF-α, thereby indicating that AEOV exerted potent anti-inflammatory effect. Biphenyldicarboxylate produced mild decrease in the level of TNF-α.

AEOV exhibited anti-inflammatory activity by the inhibition of NFκB pathway. As shown in Figure 5B, CCl_4_ injection significantly increased expression of IκB kinase α (IKKα) and phosphorylated inhibitory factor kappaB-alpha (p-IκBα), and promoted nuclear translocation of NFκB in liver tissues when compared to normal mice. Treatment with AEOV or biphenyldicarboxylate significantly reduced expression of IKKα and p-IκBα, and attenuated NFκB translocation to nuclei compared to CCl_4_-induced mice without AEOV treatment. Additionally, we performed an immunohistochemical examination using an antibody against NFκB. Data shown in Figure 5C were well consistent with western blotting analysis, demonstrating that AEOV attenuated CCl_4_-induced NFκB translocation, which may have contributed to the decreased secretion of proinflammatoty cytokine TNF-α.

### 2.5. Cytoprotective Effect of Epigoitrin against H_2_O_2_-Induced HepG2 Cells

**Figure 6:**
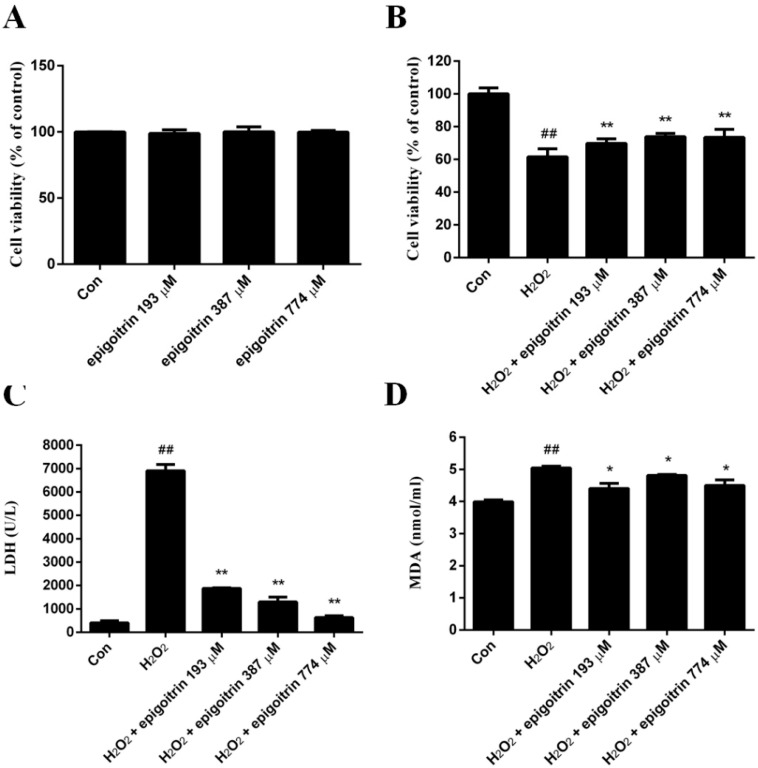
Cytoprotective effect of epigoitrin against H_2_O_2_-induced HepG2 cells. (**A**) Effect of epigoitrin on HepG2 cells viability determined by 3-(4,5-dimethylthiazol-2-yl)-2,5-diphenyltetrazolium bromide (MTT) assay. Cells were cultured with different concentrations of the epigoitrin (193, 387, and 774 µM) for 12 h. Values are the means ± SEM of three independent experiments; (**B**) Effect of epigoitrin on the viability of HepG2 cells with H_2_O_2_ exposure. Cells were incubated with H_2_O_2_ (0.4 mM, for 2 h) after pretreatment with epigoitrin (193, 387, and 774 µM) for 12 h. The cell viability was determined by the MTT assay. Values are the means ± SEM of three independent experiments. ## *p* < 0.01 versus control (untreated cells), ** *p* < 0.01 versus H_2_O_2_-treated cells. Effect of epigoitrin on lactate dehydrogenase (LDH) (**C**) and malondialdehyde (MDA) (**D**) levels in H_2_O_2_-induced HepG2 cells. Values are the means ± SEM of three independent experiments. ## *p* < 0.01 versus control (untreated cells), ** *p* < 0.01, * *p* < 0.05 versus H_2_O_2_ treated cells.

In order to evaluate the probable cytotoxicity of epigoitrin on HepG2 cells, cell viability was measured after HepG2 cells were treated with different concentrations of epigoitrin for 12 h. As shown in Figure 6A, there was no inhibitory effect on the viability of HepG2 cells after exposure to epigoitrin at the concentrations of 193–774 µM for 12 h.

Figure 6B indicates the cytoprotective effect of epigoitrin on HepG2 cells against the oxidative damage induced by H_2_O_2_. The exposure of HepG2 cells to 0.4 mM H_2_O_2_ for 2 h significantly reduced the cell viability. Pretreatment of HepG2 cells with epigoitrin at the concentrations of 193–774 µM remarkably decreased the cytotoxicity resulted from the exposure to H_2_O_2_. Notably, levels of lactate dehydrogenase (LDH) and malondialdehyde (MDA) elevated in H_2_O_2_-induced HepG2 cells, which reflected marked increase of cell death. However, when HepG2 cells were pretreated with epigoitrin, LDH and MDA levels reduced markedly, almost comparable to those of the control cells.

### 2.6. Anti-Oxidative and Anti-Inflammatory Effects of Epigoitrin against H_2_O_2_-Induced HepG2 Cells 

**Figure 7:**
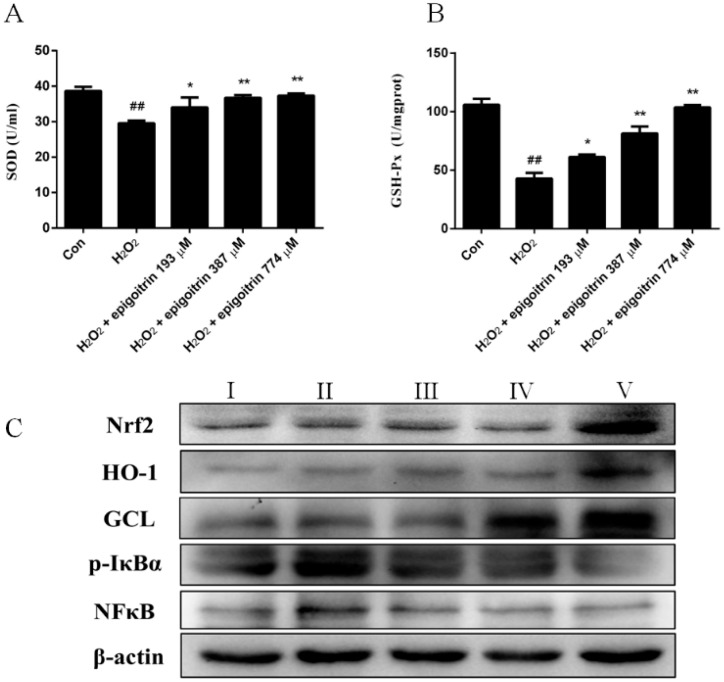
Anti-oxidative and anti-inflammatory effects of epigoitrin against H_2_O_2_-induced HepG2 cells. (**A**,**B**) Effect of epigoitrin on superoxide dismutase (SOD) and GSH-Px activities in H_2_O_2_-induced HepG2 cells. Values are the means ± SEM of three independent experiments. ## *p* < 0.01 versus control (untreated cells), ** *p* < 0.01, * *p* < 0.05 versus H_2_O_2_-treated cells; (**C**) Effect of epigoitrin on the expression of Nrf2, GCL, HO-1, p-IκBα, and NFκB in H_2_O_2_-induced HepG2 cells. Cells were treated with H_2_O_2_ (0.4 mM, for 2 h) after pretreatment with epigoitrin for 12 h. Protein expression was then detected by western blot analysis. Groups are as follows. І: saline; ІІ: saline + H_2_O_2_; ІІІ: epigoitrin 193 µM + H_2_O_2_; ІV: epigoitrin 387 µM + H_2_O_2_; V: epigoitrin 774 µM + H_2_O_2_.

As shown in Figure 7A,B, H_2_O_2_ administration significantly reduced superoxide dismutase (SOD) and GSH-Px activities in HepG2 cells compared with the control group. Epigoitrin pretreatment dose-dependently induced marked increase of SOD and GSH-Px activities . Similarly, when H_2_O_2_-induced HepG2 cells were pretreated with epigoitrin, the expression of Nrf2 and GCL appeared to continuously increase in comparison with the H_2_O_2_-treated cells (Figure 7C).

Expression of p-IκBα and NFκB was markedly elevated after HepG2 cells were treated with H_2_O_2_. With epigoitrin pretreatment, levels of p-IκBα and NFκB (Figure 7C), decreased significantly in a dose-dependent manner, indicating a significant anti-inflammatory effect of epigoitrin against H_2_O_2_-induced HepG2 cells. 

## 3. Discussion

**Figure 8:**
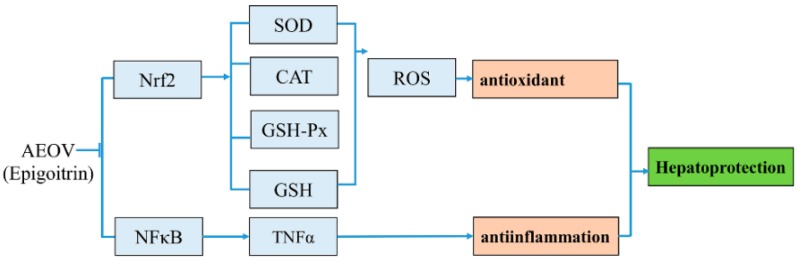
A schematic illustration of a proposed mechanism.

To the best of our knowledge, no study has been reported on the hepatoprotective effect of AEOV. Thus, the present study was performed to investigate the hepatoprotective effect of AEOV in vivo using CCl_4_-induced mouse model and evaluate the cytoprotective effect in vitro using H_2_O_2_-induced HepG2 cells, and further explore the underlying mechanism. AEOV helped to attenuate liver injury induced by CCl_4_ in a dose-dependent manner. Epigoitrin exhibited strong cytoprotective effect against H_2_O_2_-induced oxidative damage in HepG2 cells. Additional study revealed that hepatoprotective actions of AEOV and epigoitrin were largely related to their strong anti-oxidative and anti-inflammatory properties via regulation of Nrf2 and NFκB pathways (Figure 8).

Alkaloids and organic acids are two major chemical portions of AEOV. Naturally occurring alkaloids widely isolated from numerous common medicinal plants, have long been known to exert a broad spectrum of pharmacological properties, including anti-endotoxic, antiviral, anticancer, antinociceptive, anti-inflammatory, antimalarial, and antipyretic effects [19,20]. Protocatechuic acid and caffeic acid are the major organic acid constituents isolated from AEOV. They are also derived from various Chinese herbal medicines, and are reported to activate anti-oxidative genes that combat oxidative stress and cell apoptosis [21,22]. Alkaloids may chemically react with organic acids in vivo, producing a better pharmacological effect. Consequently, AEOV, a mixture of alkaloids and organic acids, holds promise as a hepatoprotective agent. In the present study, we found that AEOV pretreatment restored liver injury induced by CCl_4_ in a dose-dependent manner, and levels of ALT and AST in serum were also substantially decreased with AEOV pretreatment, firmly confirming the hepatoprotective effect of AEOV in vivo. Moreover, the cytoprotective effect in vitro was demonstrated in H_2_O_2_-induced HepG2 cells using epigoitrin, the major alkaloid compound isolated from AEOV. Previous screenings showed that epigoitrin had strong inhibitory effect on influenza virus [23]. However, here, we demonstrated the cytoprotective, anti-oxidative, and anti-inflammatory effects of epigoitrin against H_2_O_2_-induced oxidative damage in HepG2 cells.

Accumulating evidence suggests that ROS-induced oxidative stress plays a central role in several human pathologies including liver injury, causing the peroxidation of lipids, protein oxidation, DNA damage, mitochondrial dysfunction and altered signal transduction [24,25,26]. Hence, effective therapeutic approaches are urgently needed to inhibit the detrimental effect of ROS, for instance, through the induction of endogenous enzymatic antioxidants [27]. Nrf2, a basic region-leucine zipper (CNC bZip) transcription factor, plays a key role in modulating cellular defense against oxidative stress [28,29,30]. Under normal conditions, Nrf2 is retained in the cytoplasm by binding to its negative regulator, Keap1. The production of ROS typically provokes a cellular oxidative stress environment, which causes dissociation of Keap1 from Nrf2, thereby allowing Nrf2 to translocate into the nucleus [31,32], activating antioxidant genes and enzymes including glutathione peroxidase (GPx), glutathione-S-transferase (GST), heme oxygenase (HO), glutamate cysteine ligase (GCLc) and superoxide dismutase (SOD) by binding to antioxidant response elements (ARE) in the promoter regions of its target genes [33,34,35], which subsequently contributes to adaptation and protection of cells against oxidative stress [36]. In the present study, we found that AEOV enhanced nuclear translocation of Nrf2, decreased level of Keap1, and elevated expression of HO-1 and GCL, indicating involvement of the Nrf2-ARE pathway in AEOV-mediated hepatoprotective effect in vivo. Moreover, T-SOD, CAT, GSH-Px, and GSH, playing essential roles in detoxifying the reactive toxic metabolites of many toxins, were substantially elevated by AEOV treatment, confirming the anti-oxidative property of AEOV in liver tissues of CCl_4_-induced mice. In addition, activities of SOD and GSH-Px and expression of Nrf2 and GCL were also elevated significantly with epigoitrin pretreatment in H_2_O_2_-induced HepG2 cells, which indicated the anti-oxidative effect of epigoitrin in vitro.

It has been well established that an excessive inflammatory reaction, including the release of pro-inflammatory cytokine such as TNF-α, is largely responsible for liver injury [37,38,39]. The inflammatory response is mainly triggered by NFκB, a pleiotropic regulator of various genes encoding inflammatory mediators [40,41,42]. In an unstimulated state, NFκB is stabilized in cytosol by inhibitory factor κB-α (IκBα) [42]. After being activated by various inducers including cytokines, reactive oxygen species (ROS) and bacterial lipopolysaccharides, IκBα becomes phosphorylated triggering its proteolytic degradation, leading to transient translocation of NFκB from the cytoplasm to the nucleus, thus activating the expression of pro-inflammatory proteins, cytokines, chemokines, adhesion molecules or enzymes involved in the inflammatory process [43,44,45,46,47]. In the present study we found that treatment with AEOV in vivo reduced the overall nuclear localization of NFκB via reducing IKKα and inhibiting the phosphorylation and degradation of IκBα, thereby precluding the expression of NFκB target genes, rescuing cells from inflammatory injuries. Additionally, expression of p-IκBα and NFκB also decreased significantly with epigoitrin pretreatment in H_2_O_2_-induced HepG2 cells. Taken together, with our results, the hepatoprotective effect of AEOV in vivo and the cytoprotective effect of epigoitrin in HepG2 cells are specifically related to the observed anti-inflammatory effect as supported by the strong inhibition of NFκB pathway. 

In summary, we successfully demonstrated the potent protective effect of AEOV against CCl_4_-induced liver injury and confirmed the cytoprotective effect of epigoitrin in HepG2 cells. The hepatoprotective effect of AEOV in vivo and the cytoprotective effect of epigoitrin in H_2_O_2_-induced HepG2 cells were associated with their strong anti-oxidative and anti-inflammatory properties via enhancing the Nrf2 response and inhibiting the NFκB signaling pathway. Epigoitrin, as a major component of AEOV may be partially responsible for the anti-oxidative and anti-inflammatory effects of AEOV, which needs further investigation.

## 4. Methods

### 4.1. Plant Material and Extract Preparation

Seeds of *O. violaceus* were collected in Beijing province, Peopleʼs Republic of China, in August 2015. The material was identified and authenticated by Wan-Long Ding. A voucher specimen (NO. 150811) was deposited at the Institute of Medicinal Plant Development, Chinese Academy of Medical Science and Peking Union Medical College (Beijing, China). To prepare aqueous extract, the air-dried and powdered seeds of *O. violaceus* (20.0 kg) were extracted three times with water (3 × 40 L, 2 h each), then the solvent was evaporated using a rotary evaporator. The obtained extract was freeze-dried and stored at −20 °C.

### 4.2. HPLC Analysis

After extraction with water, the aqueous extract was concentrated to the small volume (3 L), and applied on a D-101 macroporous adsorptive resin (20 kg, 20 × 200 cm), eluting with H_2_O (60 L) and 10% EtOH (80 L). The 10% EtOH fraction was concentrated under reduced pressure, and the residue (400 g) was subjected to column chromatography (CC) on silica gel (100–200 mesh, 15 × 60 cm) eluting with a stepwise gradient of CH_2_Cl_2_/CH_3_OH (10:1 to 1:1, 5 L) to obtain eight fractions (Fr. A–Fr. H). Fr. F (30 g) was subjected to chromatography using ODS MPLC (ODS Medium Pressure Preparative Liquid Chromatography) elution with MeOH-H_2_O (10:90; 30:70; 50:50), yielding three fractions (Fr. F1-3). Fr. F1 (5.7 g) was separated by a Sephadex LH-20 column (5 × 80 cm), eluted with MeOH, and then purified by preparative HPLC affording compounds 1 (15.6 mg), 2 (10.2 mg), and 3 (11.8 mg). Fr. F2 (4.6 g) was separated via reverse-phase chromatography over C-18 silica gel, eluted with MeOH-H_2_O (10:90; 30:70), and then purified through preparative HPLC elution using a MeOH-H_2_O (25:75) system and a Kromasil RP-18 column. Finally, compounds 4, 5 (15.3 mg), 6, 7 (10.8 mg), 8 (11.1 mg), 9 (5.4 mg), 10 (10.1 mg), 11 (9.4 mg), 12 (8.9 mg), 13 (5.7 mg), and 14 (10.1 mg) were obtained. Their structures were elucidated by spectroscopic analysis and comparison with data in the literature.

### 4.3. Cell Culture

Human HepG2 cells were cultured in Dulbecco’s Modified Eagle Medium (DMEM) supplemented with 10% fetal bovine serum (FBS), 100 U/mL penicillin and 100 µg/mL streptomycin at 37 °C in an atmosphere of 5% CO_2_.

### 4.4. Cell Viability Evaluation

Initially, the probable cytotoxicity of epigoitrin (193 to 774 µM) on HepG2 cells was assessed using the 3-(4,5-dimethylthiazol-2-yl)-2,5-diphenyltetrazolium bromide (MTT) method. Briefly, 24 h after being seeded in 96-well plates, cells were treated with epigoitrin at different concentrations (193, 387, and 774 µM) for 12 h. Then, the medium was removed, and the cells were washed with phosphate buffered saline (PBS), followed by MTT (0.5 mg/mL) incubation for an additional 4 h. Then the MTT-formazan product was dissolved in 150 µL of dimethyl sulfoxide and the optical density of the solution was measured at 570 nm using a microplate spectrophotometer. 

Next, the cytoprotective effect of epigoitrin on H_2_O_2_-induced HepG2 cells was evaluated. Following the exposure to epigoitrin (193 to 774 µM) for 12 h, cells were washed and treated with H_2_O_2_ (0.4 mM) for another 2 h. Cell viability was then assessed by MTT method. The cells without treatment with epigoitrin or H_2_O_2_ were considered as control with cell viability of 100 %. Cell viability was determined as a percentage of viable cells of treated wells to control wells. 

### 4.5. Measurement of LDH, MDA, SOD, and GSH- Px in H_2_O_2_-Induced HepG2 Cells

HepG2 cells were pretreated by different concentrations of epigoitrin (193, 387, and 774 µM) for 12 h and then exposed to 0.4 mM H_2_O_2_ for 2 h. The supernatant was then used for LDH detection. After preparing cell lysates by repeated freeze-thaw cycles, the MDA, SOD, GSH-Px activities were evaluated in a 96-well plate using their respective activity assay kits (Nanjing Jiancheng Bioengineering Institute, Nanjing, China) following the manufacturer's instructions. 

### 4.6. Animals

Male Balb/c mice were obtained from Vital River Laboratory Animal Technology Co., Ltd. (Beijing, China). Animals were kept in a 12 h light/dark cycle under standard conditions with free access to food and water. All animal care and experimental protocols used in this study were approved by the Institutional Animal Care and Use Committee at the Institute of Medicinal Plant Development, Chinese Academy of Medical Sciences (SLXD-2015111972, 19 November 2015).

### 4.7. Induction of CCl_4_-Mediated Liver Injury

Mice were randomly divided into six different groups (*n* = 8) and orally administrated with relevant drugs for 4 days: Group I (normal control group, saline), Group II (model group, saline), Group III (AEOV 125 mg/kg), Group IV (AEOV 250 mg/kg), Group V (AEOV 500 mg/kg), and Group VI (biphenyldicarboxylate 150 mg/kg, Beijing Union Pharmaceutical Factory, Beijing, China). Subsequently, mice in Groups II-VI were given a single intraperitoneal injection of 0.1% CCl_4_ (10 µL/g, dissolved in olive oil). Meanwhile, mice in Group I were intraperitoneally injected with vehicle (10 µL/g, olive oil).

### 4.8. Liver Weight and Liver Index

Sixteen hours after CCl_4_ injection, mice were weighed and sacrificed. Livers were then collected and weighed to calculate liver index using the following formula: Liver index = liver weight/body weight × 100%

Small portions of each liver were routinely processed for histological examinations, and other portions were kept at −20 °C.

### 4.9. Serum Biochemistry

Serum ALT and AST were assayed using commercially available test kits with a biochemistry analyzer system according to the manufacturer´s instructions.

### 4.10. Assay of Reactive Oxygen Species (ROS) and Hepatic Enzymes in Liver Tissues

Livers were homogenized in nine volumes of ice-cold normal saline. The homogenate was centrifuged at 4 °C (4000 r/min, 10 min), and the resultant supernatant was used for the assay of hepatic enzymes. The activities of SOD, CAT, GSH-Px, and GSH and the level of ROS were determined using their respective activity assay kits (Nanjing Jiancheng Bioengineering Institute, Nanjing, China) following the manufacturer's instructions.

### 4.11. Histological Analysis

Liver tissues were fixed in 10% formaldehyde solution. The fixed tissues were then embedded in paraffin and cut into 3 µm sections which were then deparaffinized in xylene and rehydrated in a graded series of ethanol. The severity of liver injury was assessed by morphometric evaluation of liver slides with hematoxylin and eosin staining. 

### 4.12. Immunohistochemical Analyses

For immunohistochemistry, the sections were blocked with a buffered blocking solution (3% bovine serum albumin in phosphate-buffered saline (PBS) for 15 min. After blocking, sections were incubated with the primary antibodies for NFκB (1:50) and Nrf2 (1:50) at 4 °C overnight, followed by washing with PBS and co-incubation with a secondary antibody at room temperature for 1 h. Then, sections were visualized with 3’3-diaminobenzidine and nuclei were then counterstained with hematoxylin. The Image-ProPlus 4.5 Software (Media Cybernetics) was used to analyze the expression of proteins.

### 4.13. Western Blot Analysis

Liver tissues and HepG2 cells were homogenized in a standard RIPA buffer supplemented with a cocktail of protease and phosphatase inhibitors. The protein concentrations of the extracts were determined using a BCA (Bicin-choninic Acid) Protein Assay reagent kit (Beyotime Biotech, Nanjing, China). An equal amount of total protein per lane was fractionated on an 8% sodium dodecyl sulfate-polyacrylamide gel. After electrophoresis, the gels were transferred onto polyvinylidene difluoride membranes, which were then blocked with Tris-buffered saline containing 5% nonfat milk at 4 °C for 2 h. The membranes were incubated with primary antibodies (1:300) over night at 4 °C. After washing three times, the membranes were incubated with horseradish peroxidase (HRP)-conjugated secondary antibodies at room temperature for 1 h and subsequently processed for enhanced chemiluminescence (ECL) detection using a commercial kit (Beyotime Biotech, Nanjing, China). Signals were detected using a chemiluminescence system (Bio-Rad, Hercules, CA, USA). β-actin content was used as an internal control. 

### 4.14. Statistical Analysis

The data were presented as means ± standard error and compared by one way analysis of variance. Significant difference between groups was determined using Duncan’s test. Differences were considered to be statistically significant when *p* < 0.05.

## Figures and Tables

**Table 1:** Effect of AEOV on body weight and liver index in mice.

Group	Dose (mg/kg)	Body Weight (g)	Relative Liver Weight (g/100 g Body Weight)
Ι	-	19.7 ± 0.89	4.67 ± 0.17
II	-	20.13 ± 0.6	5.02 ± 0.22
ΙΙΙ	125	19.21 ± 0.91	4.65 ± 0.39
ΙV	250	19.68 ± 0.65	4.85 ± 0.16
V	500	19.85 ± 0.76	4.84 ± 0.22
VI	150	20.6 ± 0.8	5.13 ± 0.21

І: saline; ІІ: saline + CCl_4_; ІІІ: AEOV 125 mg/kg + CCl_4_; ІV: AEOV 250 mg/kg + CCl_4_; V: AEOV 500 mg/kg + CCl_4_; VІ: biphenyldicarboxylate 150 mg/kg + CCl_4_.
